# Healthcare professionals’ perspectives on digital biomarkers for monitoring inflammatory arthritis: insights from a qualitative study rooted in design thinking

**DOI:** 10.1016/j.ero.2025.11.021

**Published:** 2025-12-19

**Authors:** Patty de Groot, Sina Fadaei, Jolanda J. Luime, Wendy Wagenaar, Marijn Vis, Marc R. Kok, Wouter H. Bos, Ilja Tchetverikov

**Affiliations:** 1 Department of Rheumatology, Erasmus Medical Center, Rotterdam, The Netherlands; 2 Department of Rheumatology & Clinical Immunology, University Medical Center Utrecht, Utrecht University, Utrecht, The Netherlands; 3 Tranzo, Tilburg School of Social and Behavioural Sciences, Tilburg University, Tilburg, The Netherlands; 4 Department of Rheumatology and Clinical Immunology, Maasstad Hospital, Rotterdam, The Netherlands; 5 Amsterdam Rheumatology and Immunology Center, Reade Rheumatology and Rehabilitation, Amsterdam, The Netherlands; 6 Department of Rheumatology, Albert Schweitzer Hospital, Dordrecht, The Netherlands

## Abstract

**Objectives:**

The objective of this paper was to explore rheumatology healthcare professionals’ (HCPs) perspectives on the potential role, value, and implementation challenges of digital biomarkers (dBMs) for monitoring inflammatory arthritis (IA), specifically psoriatic and rheumatoid arthritis.

**Methods:**

Following the Design Thinking methodology, 7 focus groups and 1 interview were conducted with a total of 34 rheumatology HCPs. The topic guide covered: (remote) monitoring psoriatic and rheumatoid arthritis; the clinical consultation; work satisfaction; and the role of digital technologies in rheumatology. Thematic analysis followed Braun and Clarke’s methodology, supported by investigator triangulation and member checking to ensure credibility.

**Results:**

Content analysis revealed 5 overarching themes. Participants described current IA care as high quality but increasingly unsustainable, prompting HCPs to seek more efficient, digitally supported models of care delivery (theme 1). Limitations in best-practice digital care, including reduced assessment accuracy and weakened HCP-patient communication, were identified, leading to more conservative treatment decisions (theme 2). dBMs were regarded as complementary tools to enhance care efficiency and support data-driven, high-resolution remote monitoring (theme 3), although concerns were voiced about their accuracy, impact on therapeutic relationships, and HCP workload (theme 4). Adoption is influenced by trust in technology, professional values, and patient-specific factors (eg, disease complexity and preferences). Successful integration will require patient-centred seamless workflows and careful consideration of patient readiness (theme 5).

**Conclusions:**

This study highlights that although HCPs see potential in dBMs, their adoption is contingent on trust, clinical relevance, alignment with professional values, and implementation. Development efforts should prioritise robust evidence, clinician and patient engagement, and thoughtful integration into routine practice.

**WHAT IS ALREADY KNOWN ON THIS TOPIC**

• Current rheumatology care depends largely on intermittent patient self‐reports and in‐person assessments. Digital health technologies, including digital biomarkers (dBMs), are increasingly proposed as tools to mitigate projected workforce shortages. Research on health technology adoption consistently highlights the critical role of clinicians and patients in both the design and implementation phases.

**WHAT THIS STUDY ADDS**

• Guided by Design Thinking, this study captures foundational attitudes, expectations, and concerns of rheumatology healthcare professionals, which are crucial in responsible technical innovation. Our findings, supported by literature and previous research with patients, reveal a nuanced understanding of how digital health innovations intersect with clinical realities.

**HOW THIS STUDY MIGHT AFFECT RESEARCH, PRACTICE OR POLICY**

• Future dBM development should prioritise robust scientific and clinical validation, alongside active involvement of clinicians and patients in dBM and workflow design. Furthermore, seamless integration into patient‐centred care pathways must be ensured to support sustainable adoption.

Alt-text: Unlabelled box dummy alt text

## INTRODUCTION

Inflammatory arthritis (IA) is characterised by flare-ups of disease activity and periods of remission. Since the early 2000s, treat-to-target (T2T) strategies have significantly improved the outcomes of these diseases. However, frequent T2T monitoring is challenging due to rheumatology workforce shortages and increasing care demands. In response, digital-first hybrid care models that integrate remote and in-person monitoring are emerging as a potential solution [1].

Despite the growing interest in remote monitoring, current approaches predominantly rely on telephone or video consultations and electronic patient-reported outcomes (ePROs) [2]. These methods provide intermittent and often subjective assessments, thereby limiting their ability to capture real-time fluctuations in disease activity. In contrast, sensor-based digital health tools (DHTs) — that retrieve data from smartphones, smartwatches, and wearable devices — enable passive, objective, and quantifiable data collection of physiological and behavioural parameters (e.g., heart rate variability or physical activity with accelerometers) [3,4]. These so-called digital biomarkers (dBMs) could offer a continuous and fine-grained picture of disease activity, potentially enhancing precision in clinical decision-making, optimising resource allocation in rheumatology care, and improving patient outcomes and satisfaction [1,4,5].

A growing body of observational evidence supports the feasibility and validity of dBMs in IA across active, passive, and multimodal approaches [4,[6], [7], [8]]. Much of this progress builds on research in other disease areas, supported by cross-disease initiatives such as the Identify Digital Endpoints to Assess FAtigue, Sleep and acTivities of daily living (IDEA-FAST) consortium and the Digital Medicine Society (DiMe) [9,10]. Within IA, emerging studies in psoriatic and rheumatoid arthritis have shown that active smartphone tasks, such as the Patient Rheumatoid Arthritis Data from the Real World (PARADE) study's wrist motion assessment and Psorcast’s Digital Jar Open and 30-sec Walk tests, correlate with clinical measures of pain and joint tenderness [11,12], whereas passive accelerometer data links lower physical activity to higher disease activity and flares [13,14]. Multimodal approaches, exemplified by the weaRAble-patient reported outcome (PRO) study, integrate sensor-derived and patient-reported data to better capture fluctuations in disease activity [5], and complementary work associates behavioural smartphone data and nocturnal skin scratch with symptom burden [15,16]. Collectively, these studies mark early but promising evidence supporting the validity and potential clinical value of sensor-based digital endpoints in IA.

To bridge the gap between technological innovation and clinical feasibility [17], this study applies Design Thinking to explore rheumatology healthcare professionals’ (HCPs) perspectives on dBMs for disease activity monitoring of IA, focusing on psoriatic and rheumatoid arthritis care in the Netherlands [18,19]. The study complements our previous research on patient perspectives, which showed patients are receptive to the concept of dBMs, especially when proposed by their HCP [20]. Digital transformation in healthcare requires reconfiguration of workflows and systems rather than the mere integration of new tools [21]. Achieving this demands that innovators understand, respect, and design around HCPs' core needs and concerns, which necessitates their direct involvement [22]. Accordingly, this research captures clinician perspectives on clinical care, digital care, and dBMs in particular, to inform the design of dBMs that are both clinically meaningful and feasible for routine practice.

## METHODS

### Study design

**Figure 1:**
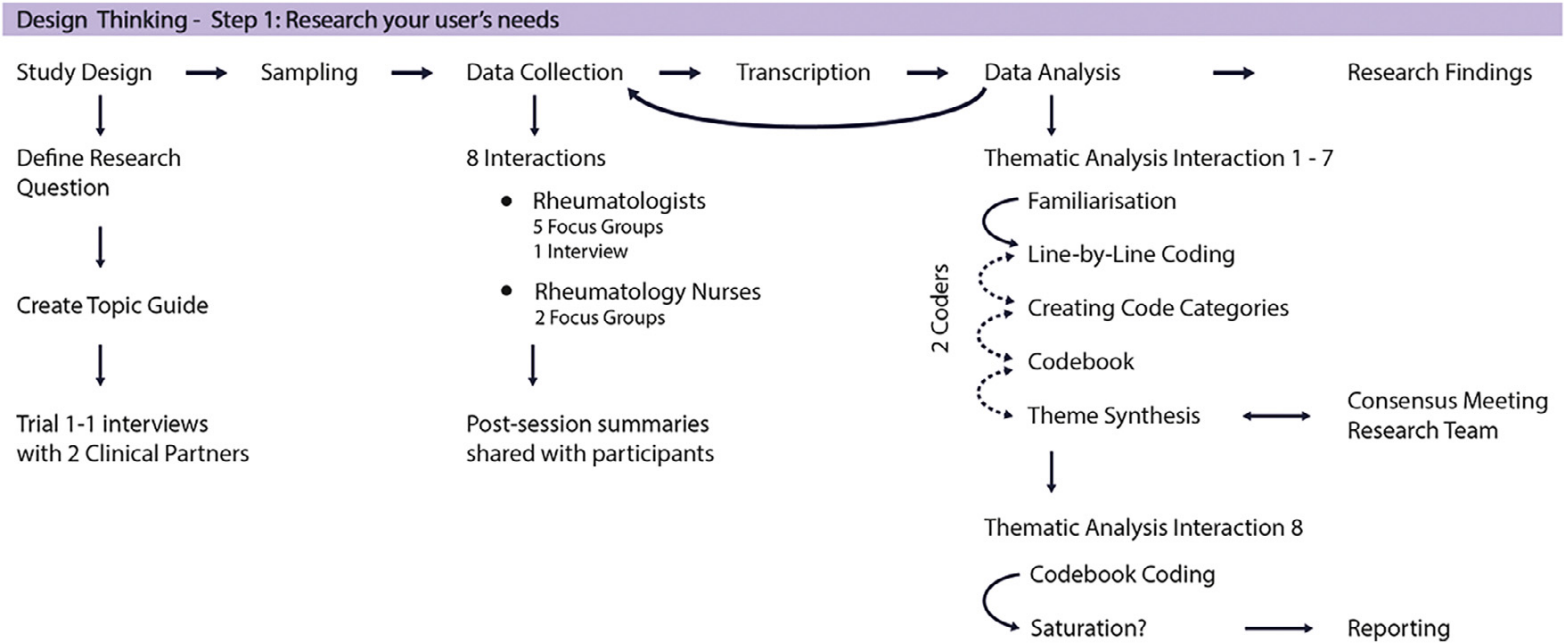
The qualitative research methodology as adopted within the ACCEPT study.

Within the framework of the Design Thinking methodology, the study adopted an exploratory qualitative design, incorporating focus groups and a single interview (Fig 1). Convenience sampling was applied where HCPs were recruited through personal invitations from the rheumatologists in our research team. HCPs were invited from a range of hospitals, including academic hospitals, general hospitals, and independent treatment centres. Focus groups were scheduled separately for rheumatologists and rheumatology nurses, with each approximately 60-minute session comprising participants from various hospitals. The online ZOOM focus groups were conducted between January and September 2022 with 1 additional focus group in February 2025 to verify our results. PdG moderated all focus groups, with alternating observers AP, LD, JJL, and SF. Interactions were audio-recorded, and field notes were taken by the observers to capture additional insights. Post-session summaries were shared with the participants to enhance the credibility of the findings.

A topic guide, underpinned by the graphical framework for clinical decision-making [23] and the theoretical domain framework/COM-B model [24], structured the data collection. It was piloted in 2 one-to-one interviews. After refinement, the topic guide covered: disease activity monitoring in psoriatic and rheumatoid arthritis; usefulness of consultations; and the concept of dBMs to measure disease activity. The topic guide was adjusted once after the fourth interaction to incorporate additional perspectives on how technology in general could contribute to the HCPs’ work (Supplement S1). Data saturation, defined as the point at which no new themes or information emerged from additional interviews or focus groups, was reached after 7 interactions, as determined by consensus within the research team and verified in 1 additional focus group.

### Analysis

The audio recordings were transcribed verbatim and pseudonymised using Amberscript software and corrected by PdG to ensure accuracy. Thematic analysis of the transcripts was performed by 2 researchers: PdG and SF. Both researchers are conducting their doctoral research in rheumatology departments and have experience with qualitative analysis. PdG (female) has a background in biomedical engineering and SF (male) in medicine. None of the researchers were acquainted with the participants before the interactions.

The transcripts were analysed following Braun and Clarke's [25] approach to thematic analysis. The researchers began by familiarising themselves with the data, reading through the transcripts line by line to identify meaningful text segments. The transcripts were then uploaded to ATLAS.ti for detailed coding, conducted independently by PdG and SF. After coding each transcript, emerging codes and themes were compared and refined. This iterative process allowed for the identification and reorganisation of themes as analysis progressed. Investigator triangulation was applied throughout, with theoretical frameworks consulted when necessary, leading to the development of a final codebook encompassing themes and subthemes. Additional triangulation sessions with a broader research team — WW (patient partner), JJL (epidemiologist), and IT (rheumatologist) — were conducted to cross-check interpretations and resolve discrepancies.

The findings presented are reported following the Consolidated Criteria for Reporting Qualitative Research checklist (Supplement S2) [26]. Direct quotations from participants illustrate the themes and support the conclusions.

## RESULTS

Insights were collected from 25 rheumatologists and 9 rheumatology nurses (68% women; aged 31-65; work experience 1-30 years) during 7 focus groups and 1 interview. Participants varied in age, work experience, professional background, and personal usage of digital health monitoring, as presented in the Table 1 and Supplement S3 for individual participant characteristics. Of the 34 HCPs, 8 participants used smartphone health applications (e.g., step counters, sleep trackers) or sports-specific applications without a wearable. Ten participants reported the additional use of wearables for fitness monitoring. Fourteen participants did not perceive the need for health tracking. All participants demonstrated sufficient digital proficiency to join the online video call.

**Table 1:** Characteristics of rheumatology HCPs participating in the study focus groups and interview

	Rheumatologists (N = 25)	Rheumatology nurses (N = 9)	Total (N = 34)
Interaction, N = 8			
Focus groups	5	2	7
1-1 interview	1		1
Female/male, N	14/11	9/0	23/11
Age (y) mean (range)	46 (32-65)	51 (36-57)	48 (32-65)
Work experience (y) mean (range)	9 (1-27)	16 (3-30)	11 (1-30)
Work setting, N			
Academic hospital	5	1	6
General hospital	17	8	25
Independent treatment centres	2	1	3
Prior experience with digital Health measurements, N^a^			
Yes, Smartwatch	7	3	10
Yes, Smartphone	6	2	8
No experience	11	3	14
Unknown	1	1	2

HCP, healthcare professionals.

a HCPs reported their personal experiences with digital health/fitness monitoring. Smartwatch usage implies the additional usage of a smartphone application to review the collected data. Smartphone numbers indicate the usage of smartphone apps without the usage of a smartwatch.

**Figure 2:**
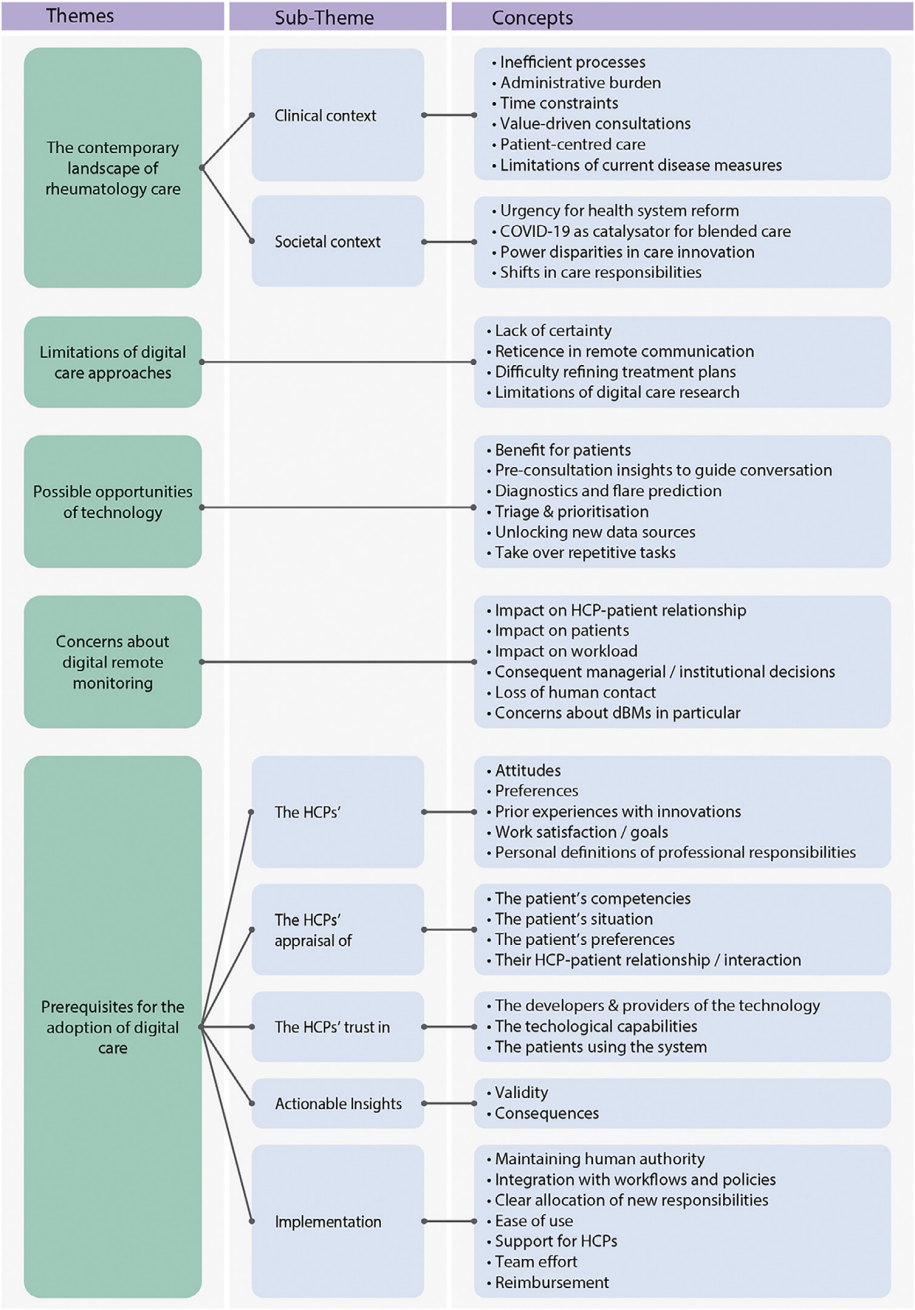
Themes, subthemes and concepts. dBM, digital biomarkers; HCP, healthcare professionals.

Content analysis of the data revealed 5 overarching themes, identified through 684 open codes, organised into 47 concepts, as indicated in Figure 2. Theme 1 describes the current landscape of rheumatology care regarding the clinical and societal context described by clinicians. Theme 2 captures the limitations experienced by HCPs when engaging in best-practice digital care. Theme 3 pertains to the perceived opportunities of digital remote monitoring technologies. Theme 4 addresses the concerns of HCPs regarding further digitalisation of chronic care. Finally, theme 5 covers the factors deemed important for adoption of digital care: HCP characteristics, appraisal of the patients’ readiness level and circumstances, as well as their trust in the technology and ensuring implementation plans are fit for clinical practice.

### Theme 1 — the contemporary landscape of rheumatology care

Although the quality of rheumatology care has improved significantly over the past decades, participants still stress the need for rigorous systemic reform. Rising demographic pressure, opposed to projected labour shortages, threatens the sustainability of the current system, forcing HCPs to seek ways for more effective and efficient delivery of care. Given the stringent time constraints faced by clinicians, the general disposition among the participants is that each consultation should be purposeful.

*We are currently in an incredibly privileged position. The current standard of care is of very high quality, but it’s not sustainable. I also think we really need to start looking: "What are we truly needed for? […]" The way we’ve organized rheumatology care now, we do a lot of check-ups, where the consultation with me doesn’t really add value […], but we do them because we’re used to it. — HCP 28*

One perspective on value generation through consultations is value to the patient, achieved by addressing and managing health-related concerns. Participants observe that younger generations of patients are increasingly more informed but also demanding. These evolving societal values are less compatible with the traditionally paternalistic model of care. To meet patients’ needs, participants wish to prioritise patient-centred discussions over engaging in routine questions or repetitive tasks.

*That’s more about authority: "If the doctor tells me to, I’ll take it and see how it goes." Whereas with the younger generation, I really see people looking critically, having read the leaflets and googled what effects it might have. And then they say: "Hmm, I’d actually like to talk about that." — HCP 18*

However, participants report spending a considerable amount of time navigating Electronic Health Records (EHRs), retrieving information, and documenting. They indicate that large EHR vendors dominate the Dutch market and obstruct data sharing by hindering system interoperability. Consequently, they find themselves spending less time on patient care and more on imposed ancillary tasks. Among participants, there is a widespread call for streamlined digital infrastructures and a reduction in administrative burden to improve the utilisation of costly health resources.

*I often think: "I can do so much more than what I’m doing now." I mostly do a lot of administration, a lot of ancillary work […], whereas I would have preferred to use that time to talk longer with my patient. — HCP 20*

Participants strive to optimise the efficiency of outpatient services by ensuring consultations are both necessary and impactful. A restructuring of care responsibilities is anticipated, where rheumatologists attend to complex cases, whereas stable patients may be redirected to alternative care models. Consultation type (e.g., in person, remote, or deferred) and frequency should align with individual patient needs. The COVID-19 pandemic accelerated blended care adoption. HCPs are considering whether to refine, structurally integrate, or scale back these temporary solutions, as care shifts back from crisis response to sustainable solutions.

*There might be a shift coming. […]. Where maybe we can monitor stable patients more remotely or through another healthcare professional […] and that we can focus more on the people who are more complex, who might need a bit more time and attention. — HCP 5*

### Theme 2 — limitations of best-practice digital care approaches

The COVID-19 pandemic accelerated the utilisation of digital care. Participants reported that assessing disease activity during remote consultations (telephone calls, video calls, or text messaging) posed a significant challenge. This difficulty stems from the inability to rely on subtle, context-rich observations that are naturally present during in-person interactions and extend beyond the physical examination (e.g., patients’ gait upon arrival, posture, or facial expressions). Instead, physicians must rely on self-reported symptoms, which often cannot be confirmed upon physical examination. Thus, without these observations, nuances that aid in distinguishing inflammatory activity from alternative sources of pain or fatigue may be overlooked. Participants fear both the consequences of under- and overestimation of active disease by their patients.

*There’s a group of people who just accept some swelling. […] That doesn’t always make it easy to assess it remotely. […]. We really need palpation. If it’s really swollen, you can see it visually, but with subtle ones. No, that’s difficult. — HCP 22*

*You can’t see it. […]. So you’re kind of dependent on what the person reports. Because it does happen that people indicate they have swelling, and then you invite them to the clinic and it turns out they don’t. — HCP 16*

Good communication was emphasised as a key factor in building patient-HCP trust, which in turn is a critical factor in therapy adherence. Participants reported on communication barriers during remote interactions. They find it more difficult to convey genuine interest in their patients and feel that patients may downplay concerns during remote conversations.

*The human aspect, genuinely showing interest in the patient. I personally find that more difficult when I’m calling someone and […] how you’re trusted also depends on that. In the long term, it also affects the success of your therapy […] if they eventually trust you and follow your advice. — HCP 2*

Several participants mentioned a ‘clinical instinct’ during in-person consultations that helps identify discrepancies between patients’ reports and how they present physically. Without the confidence afforded by in-person consultations, they are more cautious with therapy adjustments. This tendency towards more conservative clinical decisions, combined with the inability to detect subtle signs of inflammation remotely, complicates the precise tailoring of the medication regimens to the individual patient.

*What I’ve noticed is that during telephone consultations, you’re less inclined to adjust medication dynamically, especially to taper it, for example. Because you tend to think: “Well, it’s going fine, let’s just keep it as is.” When you see someone in person, you’re more likely to say: “It’s going well, I’ll notice next time if anything changes.” — HCP 22*

### Theme 3 — possible opportunities of technology

Participants perceive remote monitoring technology as an inevitable development with potential benefits to both patients and care providers.

According to the participants, technology offers an opportunity to collect vast amounts of data previously inaccessible. Novel multimodal datasets from the EHR, patient reported outcome mesaures (PROMs), wearables, or other sources (e.g., grocery store loyalty cards) could provide deeper insights into patient health and disease progression. They could contribute to symptom objectification, novel research, and personalised interventions, including those aimed at lifestyle.

*The question is whether you can measure it differently. […]. So that you can see whether someone has active disease based on, e.g., how much or how fast they walk. And if there’s a deviation, you start thinking in completely different ways about how to measure IA and disease activity. — HCP 31*

*The pressure is to connect the right pieces of information from a huge amount of data about people. Maybe we need to link one piece of data from Albert Heijn to one thing from a sensor, one biometric variable, and one item from a questionnaire, and then we get a complete picture of what’s going on. — HCP 30*

Participants expect to gain valuable insights into patients' day-to-day health beyond traditional clinic visits. dBM metrics could provide an objective measure of disease activity that could reduce the reliance on patients’ recollection of symptoms, especially during remote consultations. Secondly, these insights could aid in triage and prioritisation of patients, ensuring that clinical visits are allocated based on real-time patient needs rather than routine scheduling. Lastly, identification of patterns that signal an increase in disease activity could potentially allow for timely intervention or even prevention of disease exacerbation.

*I really like the idea from #34: "That your smartwatch can...’. They could already see in advance where the flare was coming. […]. That you can see a value rising, even as a patient, in your own dashboard. And then the patient could think: "[…] I need to consult my rheumatologist about what’s needed." And then we would only see patients at moments when therapy actually needs to be adjusted. Hearing it like that, I thought it was a really nice vision; that there is something that measures the patient regularly, frequently, and with minimal effort. — HCP 31*

Moreover, tracking symptom changes could help cope with the time constraints of consultations. Providing clinicians with prior insights into the disease course can foster a more focused and productive consultation. Presenting the data to patients could also serve as a stepping stone for discussing certain (sensitive) topics. Moreover, HCPs state technological advances should take over repetitive tasks and relieve their administrative burden, enabling even more patient-centred care.

*If you already know something about the disease activity before someone comes in, you can get to the core a bit faster. I think that makes the conversation more substantive. […]. "Is it active disease we need to address, or is something else going on?" And that way, you could help a patient get better more quickly, whether through medication or other advice. — HCP 35*

Providing patients with access to real-time health data and discussing this data in the clinic could cultivate awareness and encourage proactive involvement in managing their chronic condition. Moreover, other tools such as chatbots could aid in on-demand patient support and education by providing trustworthy information in a format catered to patients, e.g., digital person, text, or audio.

*When someone says: "I can’t even walk 10 meters." I always find it remarkable that they can still walk out of the clinic. But then you can show them: “Yes, but you’ve taken quite a few steps.” That can be interesting. — HCP 8*

*I think technology can support us because it supports the patient and reminds them: "it’s time to get your blood checked again", "to order your pills", etc. […]. Technology can also help us provide on-demand information for the patient. — HCP 28*

### Theme 4 — concerns about digital remote monitoring

Participants expressed reservations about the adoption of dBMs, particularly concerning their impact on therapeutic relationships, their patients, and routine practice.

Concerns were expressed about future doctor-patient relationships. A patient’s character traits are markedly more challenging to discern remotely but are important to build rapport and tailor treatment plans. Remote measurement and altered communication may cause patients to feel less heard and understood, potentially undermining their confidence in their healthcare providers. This reduced sense of trust in their HCP may affect treatment efficacy, as patients may be less likely to follow medical advice.

*That’s where my concern lies; if everything becomes much more measurable and so on, how do you keep sight of who a person is?* — *HCP 33. I think maybe the personality […] gets a bit lost. Someone might come in a bit awkwardly or be wearing a strange coat, you don’t see any of that. […]. Yet it tells you something about the patient’s character, which might be useful later on when things get more difficult. — HCP 32*

Regarding patient well-being, participants cautioned that the introduction of wearables could inadvertently trigger excessive health monitoring and heightened preoccupation with chronic conditions. Constant observation could elicit guilt in patients over occasional lapses in health behaviours (e.g., being less active on vacation), posing a psychosocial burden. Additionally, there is the apprehension that if you do not touch base with your patients periodically, issues such as medication mismanagement, active disease in stoic patients, or gradual disengagement from care may go unnoticed, ultimately compromising long-term health outcomes.

*A downside, in my view, could be: to what extent will people be constantly focused on their chronic condition? — HCP 9. If they have a lazy day for once, the step counter records that. […]. Is that a motivator, or might people feel ashamed? — HCP 17*

*How often I encounter that it turns out people don’t use their medication properly. […]. I see it in everyone, even the experienced ones who think: “well, I’ll just doctor myself.” But also the newer patients who don’t understand it. — HCP 18*

A final source of hesitation relates to the HCPs' day-to-day work. Despite dBMs being positioned as tools to alleviate workload and redirect focus to patients, participants are not fully convinced. Firstly, they foresee additional tasks through notifications (based on noisy dBM signals) that require review, interpretation, and follow-up with patients. Secondly, misuse or misunderstanding of digital systems by patients may result in additional work to rectify misconceptions. And thirdly, participants use check-ins with stable patients to recuperate, catch up with tasks, and make up for lost time. It is noted that a schedule consisting exclusively of complex patients would be regarded as a significant workload increase.

*I do think it will give us more insights. The question is, though; I also foresee it creating more work. Because we have to go through the data, and we also have to be able to interpret it properly. Who’s going to do that, and how do we filter out the errors? […] Every technology is prone to errors. If suddenly there are high scores because something was transmitted incorrectly, we get stressed […]. How do you deal with things like that? — HCP 13*

### Theme 5 — factors influencing the adoption of digital remote monitoring

**Figure 3:**
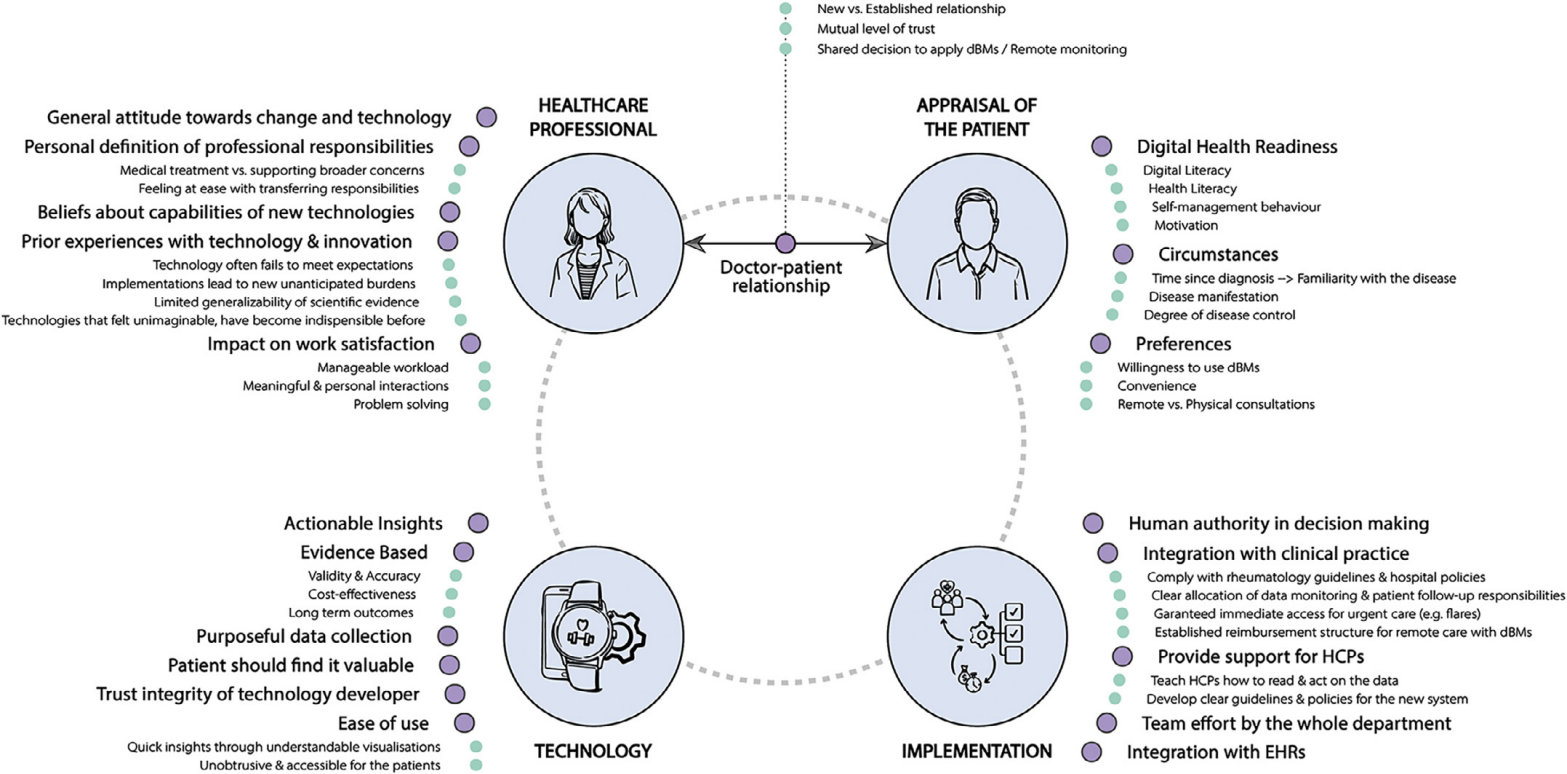
Factors contributing to rheumatology HCP willingness to adopt remote monitoring technologies. dBM, digital biomarkers; HCP, healthcare professionals, EHR; Electronic Health Records.

The various factors that influence participants' adoption of new technologies or innovations were positioned within 4 conceptual pillars, as illustrated in Figure 3.

The first pillar captures the HCPs themselves. Individual attitudes towards disruptive innovations dictate willingness to engage with new remote monitoring technologies. These individual attitudes seem to be shaped by interests, prior experiences, and beliefs. Based on experiences with disruptive innovations, participants have adopted either cautious or excited attitudes about future technological opportunities. Cautiousness was mostly expressed if work satisfaction (requiring problem solving, meaningful interactions, and diversity) was affected by past innovations. Moreover, participants differed in how they defined their professional responsibilities, and consequently, some felt more at ease transferring responsibilities than others.

*I think it is comparable to transitioning from paper records to electronic records. Yes, of course, some were enthusiastic about it, while others resisted for a long time, but now we all work with computers instead of handwritten progress sheets. So naturally, some people lead the way while others prefer to stick to tradition, but ultimately, I believe we all have to move in that direction. — HCP 5*

The second pillar captures prerequisites related to the measurement technologies. High-quality scientific studies should present compelling evidence that dBMs are reliable disease indicators. These studies should additionally demonstrate the preservation of long-term health outcomes and superior cost-effectiveness over nontechnological care delivery. In addition, participants are aware that patient engagement is one of the core elements of DHT success. Therefore, dBMs should provide actionable insights (e.g., insights into health behaviours, treatment effects, prognosis), meaning that the established indicators should contribute to the decision-making processes of both the HCP in clinic and their patients at home.

*First, I need to know what kind of information we will be receiving. […] Are these predictors of a flare? And what is the actual evidence? Perhaps I will receive an alert in HIX: "Mr X has an 80% chance of a flare in the next two weeks." Right. But then what? — HCP 34*

*So far, the figures regarding what smartphones can measure have largely been provided by the manufacturers, and – of course – those look impressive. But we will likely be handed a HIX-style computer model, validated by some engineer, and simply be told: "Here you go, use it. It’s absolutely fantastic. It was tested on 30 patients and worked just fine." [Sarcastic] — HCP 26*

The third pillar captures the necessary conditions for integrating evidence-based, easy-to-use, dBMs into clinical practice. One should carefully consider how to incorporate dBMs in clinical practice. New guidelines and policies should address best practices for dBMs with regard to task allocation, data monitoring, and clinical decision-making. Furthermore, deploying remote care would require a careful redesign of current care logistics to guarantee immediate access in case of flare. It is imperative that these changes are enforced by the entire care team. Hence, it is emphasised that the HCP, in shared decision with their patient, should have the authority whether the dBM is deployed.

*I think what most people need […] is the ability to raise alarm if something happens and be seen straight away. And if you provide those safety nets, they’ll know: "I feel comfortable being away longer, but if something happens, I can always be seen." — HCP 25*

The fourth pillar depicts which patients fit the use of dBMs. For participants to be at ease with remote monitoring, there should be a positive appraisal of the patients’ digital health readiness and their circumstances. When circumstances (e.g., diagnosis, disease control, comorbidities, times since diagnosis, etc.) permit alternative monitoring, participants should trust that patients are able to manage their disease, able to interact with the technology as intended, and that they will reach out in case of an exacerbation regardless of the dBM. Therefore, participants stress that they should familiarise themselves with their patients to establish trust and rapport before deciding to deploy remote care.

*Not everyone is equally proficient with digital tools, and whether someone is 35 or 80 makes no difference. […] We are also considering people with low literacy levels.* — *HCP 6. Indeed, not all patients are suited to this. First, they must be able to understand whether their symptoms are due to inflammation or something else; they need to have insight into their condition, and one case of RA is not the same as another. […] It is difficult to monitor everyone in a standardised way via digital methods. — HCP 5*

*I think this is especially relevant when dealing with a familiar patient population. I recently took over part of a colleague’s patient group, some of whom I had never encountered before. Naturally, I have no personal sense of them, and vice versa. That means you will overlook those patients who never complain but then suddenly turn up, fully active once again. — HCP 5*

## DISCUSSION

This qualitative study sought to understand rheumatology HCPs’ perspectives on the growing role of digital tools and emerging technologies in clinical practice. The study forms part of a Design Thinking approach [18,19] and complements prior research on patient perspectives [20]. Findings highlight clinical relevance, maintaining quality of care, and feasibility for routine practice as core elements to real-world adoption. The identified key attitudes, expectations, and concerns can help guide the development of dBMs for IA.

Participants agreed that valuable dBMs must support sustainable care by enabling trustworthy remote disease monitoring, facilitating timely and personalised interventions, and fostering a more focused and productive consultation. Feasibility hinges on seamless workflow integration, avoiding additional administrative tasks or compromising consultation time, reimbursement policies of digital care, technical interoperability of data systems, and preserving therapeutic relationships by allowing clinicians flexibility to determine whether dBMs are appropriate for their patients.

Attitudes differed considerably across participants. In line with existing research, our findings suggest that beyond validation and usability, HCPs’ receptivity may be shaped by personal beliefs about professional identity, responsibility, and technological possibilities [27]. Scepticism towards current evidence — often industry-led, small scale, and not reflective of clinical complexity — undermines their trust. The critique that digital health research lacks methodological robustness and stakeholder relevance also surfaces in the literature [[28], [29], [30]]. Participants cautioned that without robust, stakeholder-centred research, dBMs risk exacerbating workload rather than alleviating it.

Although remote care and telemedicine are acknowledged as strategic priorities, participants remain wary of compromising assessment accuracy and patient rapport. These hurdles have previously been reported as prominent concerns of telemedicine among the rheumatology community [31] and were also posed by patients [20]. Although dBMs may redistribute responsibilities between HCPs, patients, and technology, they should ultimately serve as an extension of clinicians’ expertise [1,32]. Our participants envisage dBMs as complementary tools: continuous, high-resolution data streams to detect flares, schedule in-person visits by clinical need, and support remote treatment decisions. For success, insights must be interpretable, actionable, and surface at the appropriate time.

Our results reaffirm previously identified concerns that narrowing clinical focus to easily measurable parameters could come at the expense of broader patient well-being [20,33]. The intensive effort required to develop and validate dBMs at the individual level was stressed by participants. Currently, much of the dBM evidence in rheumatology is early stage [5,6,8,12,14]. Various conceptual frameworks have been proposed to guide the transition from proof-of-concept to fit-for-purpose dBMs [1,4,34,35]. They advocate stakeholder engagement as well as continuous validation and re-evaluation, and warn against simply converting traditional clinical assessments to digital formats.

This study has limitations. Being an explorative qualitative study, findings are inherently context-specific. Although convenience sampling was applied, the sample included participants with varying characteristics (exposure to digital health included). Additionally, participants reflected on a hypothetical future, since no clinically validated dBMs exist, and rapid technological advances (e.g., generative AI, such as ChatGPT, since 2022) may have altered perceptions. The generalisability of perceptions may thus be limited once dBMs become actually clinically available. Nevertheless, enduring issues around clinical utility, evidence trustworthiness, and therapeutic relationships remain central to digital health innovation [21,32].

The eventual adoption of dBMs in clinical care will require a rigorous systemic redesign of healthcare processes from a service design [36] as well as a network perspective [37]. Rheumatology HCPs will play a pivotal role in this transition: interpreting dBM insights within the clinical context, determining when digital monitoring is appropriate, and integrating remote data into shared decision-making. Technically, EHRs continue to function as isolated data silos that are not optimised for AI-driven analytics. The integration of interoperable data, including real-world data from the EHR, wearables, ePROs, imaging, and blood sampling, is necessary for the expansion and adoption of remote disease activity monitoring [38]. Procedurally, new care models should be created by reshaping care pathways, redistributing responsibilities among HCPs, patients, and technology, and the creation of reimbursement structures that cover digital patient monitoring [39,40]. An anticipated vision is outlined by Knitza et al. (2024) with their digital-first hybrid stepped care model [1].

A gradual transition towards digital-first hybrid care will require sustained effort. The path forward involves generating robust evidence in controlled settings, followed by stepwise integration into clinical practice. This approach will help build trust and familiarity among both clinicians and patients. As technical capabilities and user acceptance grow, dBMs may take on greater monitoring responsibilities. Current advances in Large Language Model (LLM) based chatbots will further augment digital remote care as an educational tool, triage, and decision support system. Ongoing evaluation of performance, user experience, and clinical relevance will be essential to ensure that dBMs enhance, rather than disrupt, the quality and humanity of rheumatology care.

In conclusion, rheumatology HCPs are open to adopting dBMs provided these tools demonstrate clear clinical value and rest on rigorous, scientific evidence. Thoughtful integration through System Design that preserves clinician autonomy, patient trust, and workflow efficiency will be critical. Continued collaboration among developers, clinicians, and patients, which is at the core of Design Thinking, is essential to ensure dBMs meet real-world needs and enhance care delivery.

## Competing interests

PdG, SF, WW, WHB, MRK, and JJL have no disclosures. IT has been paid as a speaker for Eli Lilly, Pfizer, and UCB. MV has received grants/research support from Eli Lilly, Novartis, and UCB; and has been paid as a speaker for AbbVie, Eli Lilly, Novartis, Pfizer, and UCB.

## Appendix Supplementary materials

Image, application 1

Image, application 2

Image, application 3

Image, application 4

## Footnotes

Supplementary material associated with this article can be found in the online version at doi:10.1016/j.ero.2025.11.021.
