# Supplementary material for: Healthcare professionals’ perspectives on digital biomarkers for monitoring inflammatory arthritis: insights from a qualitative study rooted in design thinking
Source: EULAR Rheumatol Open. 2025 Dec 19;2(1):19–28. doi: 10.1016/j.ero.2025.11.021 (PMC13292230; doi:10.1016/j.ero.2025.11.021)
Supplement: Supplementary file 1 [file mmc1.docx]

**Supplementary Material S1: Focus Group Topic Guide**

| English |
| --- |

## Welcome

Welcome to our focus group on the challenges and opportunities in the current approach to disease monitoring for RA and PsA. We're really grateful that you've taken the time to help us with our research.

- Introduce Moderator & Observer(s)
- Outline the set up of the focus group to set expectations.
- Discuss Focus Group Rules
- Explain Zoom functions, e.g. raise hand

## Introduce the topic

Welkom bij onze focusgroep betreffende de pijnpunten en kansen in de huidige manier van ziektemonitoring bij RA & PsA. Super fijn dat jullie tijd vrij hebben willen maken om ons te helpen bij ons onderzoek.

Inflammatoire artritis is een langdurige ziekte waarvoor patiënten regelmatig voor een consult naar het ziekenhuis moeten komen. Patiënten geven aan dat deze consulten tijdrovend zijn en wanneer hun ziekte activiteit laag is, misschien niet altijd nodig.

Wij willen kijken of het op afstand monitoren van ziekteactiviteit toepasbaar zou zijn op de behandeling van Artritis Psoriatica & Reumatoïde Artritis. We zitten daarbij in de beginnende fase en hebben dus nog geen manier bedacht om dit te doen, maar in velden buiten de Reumatologie worden er methodes ontwikkeld die misschien ook binnen de reumatologie toegepast zouden kunnen worden. Wel vermoeden we dat om patiënten op afstand te kunnen monitoren, we ziekteactiviteit anders zullen moeten gaan meten.

We willen graag onze vinger leggen op de pijnpunten in huidige methodes van ziektemonitoring, en wat voor kansen remote monitoring kan brengen, mits het aan jullie voorwaarden kan voldoen. Hiervoor willen we met jullie sparren over de reumazorg van de toekomst en de eventuele rol van technologie daar in. We zijn hierbij specifiek gericht op de ziektebeelden Reumatoïde Artritis en Artritis Psoriatica.

## Introduce the participants

Introduction of the participants by means of 4 questions:

- Who are you?
- Where are you employed?
- How long have you been in your profession?
- Which word comes to mind first when you think of Psoriatic Arthritis?
- Consent

## Discussion

*Version 1*

1. Disease activity in RA and PsA what does it entail?
   1. How accurately do you think you are able to estimate the patients disease activity in between the visitations?
   2. How do you determine the patients disease activity?
2. How important is it to meet with your patient in-person?
   1. In your opinion, when has a consultation been useful? What is the main goal of a consultation?
   2. How do physical consultations compare to remote consultations?
3. Which information about your patient is needed to make treatment decisions?
   1. Why is this information leading in your decision making process?
4. If I were to tell you that there is a technology available that can measure disease activity of RA and PsA patients at home. These patients can visit their rheumatologist if their disease flares up or if they have a specific health related question. What would be your first thought?

*Examples of the technologies: Step counter, Activity Tracker, Smart Keypad*

- 1. Do you see any benefits?
  2. Do you see any drawbacks?
  3. Would you want try-out this technology?
  4. How do you think such a technology would affect the work that you do?
  5. What is needed to convince you about the added value of this technology?
  6. Are there any specific patient populations for which you want to apply this technology?
     - How capable do you find patients in noticing a flare and acting by contacting the hospital in time?
     - Which patients are good at this and which aren’t?
  7. How would these technologies influence your treatment decision making?

*Version 2*

1. How could we make your work easier and more fun?
   1. If you would be allowed to change the way you work, but you would have to see the same amount of patients with the same hours a week and the same number of colleagues. How would you want to change things for the better?
   2. Do you think there are any technologies available that could help you with this?
      - What would you want for this technology to help you with?
      - What kind of data would you like for this technology to gather?
      - Wat kind of benefits could this data bring?
   3. What should always remain within your job? And what would you like to put down the drain as soon as possible?
2. If I were to tell you that there is a technology available that can measure disease activity of RA and PsA patients at home. These patients can visit their rheumatologist if their disease flares up or if they have a specific health related question. What would be your first thought?

*Examples of the technologies: Step counter, Activity Tracker, Smart Keypad*

- 1. Do you see any benefits?
  2. Do you see any drawbacks?
  3. Would you want try-out this technology?
  4. How do you think such a technology would affect the work that you do?
  5. What is needed to convince you about the added value of this technology?
  6. Are there any specific patient populations for which you want to apply this technology?
     - How capable do you find patients in noticing a flare and acting by contacting the hospital in time?
     - Which patients are good at this and which aren’t?
  7. How would these technologies influence your treatment decision making?

## Close

- Shortly summarize what has been discussed.
- Ask each participant if there is anything they would like to add
- Explain what is going to happen with the provided information.
- Explain how they shall receive feedback about the results of this study
- Ask if there are any further questions or comments

| Dutch |
| --- |

*Versie 1*

1. Ziekteactiviteit bij RA en PsA – wat houdt dat in?
   1. Hoe goed denk je dat je in staat bent om de ziekteactiviteit van een patiënt in te schatten tussen de consulten door?
   2. Hoe bepaal je de ziekteactiviteit van een patiënt?
2. Hoe belangrijk is het om je patiënt fysiek te zien?
   1. Wanneer vind jij dat een consult nuttig is geweest? Wat is volgens jou het hoofddoel van een consult?
   2. Hoe verhouden fysieke consulten zich tot consulten op afstand?
3. Welke informatie over je patiënt heb je nodig om behandelbeslissingen te nemen?
   1. Waarom is deze informatie leidend in jouw besluitvorming?
4. Welke informatie over je patiënt heb je nodig om behandelbeslissingen te nemen?
   1. Waarom is deze informatie leidend in jouw besluitvorming?
5. Stel dat er een technologie bestaat waarmee patiënten met PsA thuis hun ziekteactiviteit kunnen meten. Deze patiënten kunnen dan naar de reumatoloog komen bij een opvlamming of als ze een specifieke gezondheidsvraag hebben. Wat zou je eerste gedachte daarbij zijn?

*Voorbeelden van technologieën: stappenteller, activiteitsmeter, slimme toetsenborden*

- 1. Zie je voordelen?
  2. Zie je nadelen?
  3. Zou je deze technologie willen uitproberen?
  4. Hoe denk je dat zo’n technologie jouw werk zou beïnvloeden?
  5. Wat is er nodig om jou te overtuigen van de meerwaarde van deze technologie?
  6. Zijn er specifieke patiëntengroepen waarvoor je deze technologie zou willen inzetten?
     - Hoe goed vind je dat patiënten in staat zijn om een opvlamming te herkennen en op tijd contact op te nemen met het ziekenhuis?
     - Welke patiënten kunnen dit goed en welke minder goed?

*Versie 2*

1. Hoe kunnen we jouw werk makkelijker en leuker maken?
   1. Stel dat je de manier waarop je werkt zou mogen veranderen, maar je moet wel hetzelfde aantal patiënten blijven zien, met hetzelfde aantal uren per week en met hetzelfde aantal collega’s. Wat zou je dan willen veranderen om het beter te maken?
   2. Denk je dat er technologieën zijn die je hierbij zouden kunnen helpen?
      - Waarmee zou je willen dat deze technologie je helpt?
      - Welke data zou je willen dat deze technologie verzamelt?
      - Welke voordelen zou deze data kunnen opleveren?
   3. Wat moet er volgens jou altijd onderdeel blijven van je werk? En wat zou je het liefst zo snel mogelijk willen afschaffen?
2. Stel dat er een technologie bestaat waarmee patiënten met PsA thuis hun ziekteactiviteit kunnen meten. Deze patiënten kunnen dan naar de reumatoloog komen bij een opvlamming of als ze een specifieke gezondheidsvraag hebben. Wat zou je eerste gedachte daarbij zijn?

*Voorbeelden van technologieën: stappenteller, activiteitsmeter, slimme toetsenborden*

- 1. Zie je voordelen?
  2. Zie je nadelen?
  3. Zou je deze technologie willen uitproberen?
  4. Hoe denk je dat zo’n technologie jouw werk zou beïnvloeden?
  5. Wat is er nodig om jou te overtuigen van de meerwaarde van deze technologie?
  6. Zijn er specifieke patiëntengroepen waarvoor je deze technologie zou willen inzetten?
     - Hoe goed vind je dat patiënten in staat zijn om een opvlamming te herkennen en op tijd contact op te nemen met het ziekenhuis?
     - Welke patiënten kunnen dit goed en welke minder goed?
