# Supplementary material for: Healthcare professionals’ perspectives on digital biomarkers for monitoring inflammatory arthritis: insights from a qualitative study rooted in design thinking
Source: EULAR Rheumatol Open. 2025 Dec 19;2(1):19–28. doi: 10.1016/j.ero.2025.11.021 (PMC13292230; doi:10.1016/j.ero.2025.11.021)
Supplement: Supplementary file 2 [file mmc2.docx]

**Supplementary Material S2: COREQ (Consolidated criteria for Reporting Qualitative research) Checklist**

| Topic | ItemNo. | Guide Questions/Description | Reported in Section |
| --- | --- | --- | --- |
| Domain 1: Research Team and Reflexivity | | | |
| Personal Characteristics | | | |
| Interviewer/facilitator | 1 | Which author/s conducted the interview or focus group? | PdG moderated all focus groups, with alternating observers AP, LD, JL and SF. – Methods |
| Credentials | 2 | What were the researcher’s credentials? E.g. PhD, MD | Thematic analysis of the transcripts was performed by two researchers PdG and SF. Both researchers are working on their doctoral thesis within rheumatology departments and have experience in qualitative analysis. PdG (female) has a background in biomedical engineering and SF (male) in medicine. – Methods |
| Occupation | 3 | What was their occupation at the time of the study? |  |
| Gender | 4 | Was the researcher male or female? |  |
| Experience and training | 5 | What experience or training did the researcher have? |  |
| Relationship with Participants | | | |
| Relationship established | 6 | Was a relationship established prior to study commencement? | None of the researchers were acquainted with the participants prior to the interactions. – Methods  At the beginning of each focus group the moderator and observers gave a short introduction about themselves (occupation, background), the main aim of the research and the ground rules to create a safe and confidential environment. To facilitate a dynamic discussion between the individual participants, sufficient time was given for the participants to familiarize with each other. – Supplementary Material S1: Topic Guide |
| Participant knowledge of the interviewer | 7 | What did the participants know about the researcher? e.g. personal goals, reasons for doing the research |  |
| Interviewer characteristics | 8 | What characteristics were reported about the interviewer/facilitator? |  |
| Domain 2: Study Design | | | |
| Theoretical Framework | | | |
| Methodological orientation and theory | 9 | What methodological orientation was stated to underpin the study? | The transcripts were analysed using Braun and Clarke's approach to thematic analysis (12). – Methods |
| Sampling | 10 | How were participants selected? | Convenience sampling was applied where HCPs were recruited through personal invitations from the rheumatologists in our research team. HCPs were invited from a range of hospitals, including academic hospitals, general hospitals and independent treatment centres. – Methods |
| Method of approach | 11 | How were participants approached? |  |
|  |  | Continue on next page |  |
| **Topic** | Item **No.** | **Guide Questions/Description** | **Reported in Section** |
| Sample size | 12 | How many participants were in the study? | [See item 16] |
| Non-participation | 13 | How many people refused to participate or dropped out? Reasons? | Non-participation was not tracked in this study. |
| Setting | | | |
| Setting of data collection | 14 | Where was the data collected? | Focus groups were scheduled separately for rheumatologists and rheumatology nurses, with each ~60 minute session comprising participants from various hospitals. The online ZOOM focus groups were conducted between January and September 2022 with one additional focus group in February 2025 to verify our results. – Methods |
| Presence of  non-participants | 15 | Was anyone else present besides the participants and researchers? | [See item 1] |
| Description of sample | 16 | What are the important characteristics of the sample? | Insights were collected from 25 rheumatologists and 9 rheumatology nurses (68% Female; aged 31-65; work experience 1-30 years) during 7 focus groups and one interview. Participants varied in age, work experience, professional background and personal usage of digital health monitoring, as presented in Table 1 and online supplement S3 for individual participant characteristics. Out of the 34 HCPs, 8 participants used smartphone health apps (step counters, sleep) or sports specific apps without a wearable. 10 participants reported the additional use of wearables for fitness monitoring. 14 participants did not perceive the need for health tracking. All participants possessed the digital competence to join the online video call. – Results |
| Data Collection | | | |
| Interview guide | 17 | Were questions, prompts, guides provided by the authors? Was it pilot tested? | A topic guide, underpinned by the graphical framework for clinical decision making (9) and the theoretical domain framework/COM-B model (10), structured the data collection. It was piloted in two one-to-one interviews. After refinement the topic guide covered: disease activity monitoring in psoriatic and rheumatoid arthritis; usefulness of consultations; and the concept of dBMs to measure disease activity. The topic guide was adjusted once after the 4th interaction to incorporate additional perspectives on how technology in general could contribute to the HCPs’ work (online supplement S1). – Methods |
| Repeat interviews | 18 | Were repeat interviews carried out? If yes, how many? | [See item 16] |
| Audio/visual recording | 19 | Did the research use audio or visual recording to collect the data? | Interactions were audio-recorded and field notes were taken by the observers to capture additional insights. – Methods |
| Field notes | 20 | Were field notes made during and/or after the interview or focus group? | [See Item 14] |
|  |  | Continue on next page |  |
| **Topic** | Item **No.** | **Guide Questions/Description** | **Reported in Section** |
| Duration | 21 | What was the duration of the interviews or focus group? | [See Item 14] |
| Data saturation | 22 | Was data saturation discussed? | Data saturation, defined as the point at which no new themes or information emerged from additional interviews or focus groups, was reached after 7 interactions, as determined by consensus within the research team and verified in one additional focus group. – Methods |
| Transcripts returned | 23 | Were transcripts returned to participants for comment and/or correction? | Post-session summaries were shared with the participants to enhance the credibility of the findings. – Methods |
| Domain 3: Analysis and Findings | | | |
| Data Analysis | | | |
| Number of data coders | 24 | How many data coders coded the data? | [See item 2-5] |
| Description of the coding tree | 25 | Did authors provide a description of the coding tree? | Content analysis of the data revealed 5 overarching themes, identified through 684 open codes, organised into 47 concepts as indicated Figure 2. – Results |
| Derivation of themes | 26 | Were themes identified in advance or derived from the data? | The transcripts were analysed using Braun and Clarke's approach to thematic analysis (12). The researchers began by familiarizing themselves with the data, reading through the transcripts line-by-line to identify meaningful text segments. The transcripts were then uploaded to ATLAS.ti for detailed coding, conducted independently by PdG and SF. After coding each transcript, emerging codes and themes were compared and refined. This iterative process allowed for the identification and reorganization of themes as analysis progressed. Investigator triangulation was applied throughout, with theoretical frameworks consulted when necessary, leading to the development of a final codebook encompassing themes and subthemes. Additional triangulation sessions with a broader research team – WW (patient partner), JL (epidemiologist), and IT (rheumatologist) – were conducted to cross-check interpretations and resolve discrepancies. – Methods |
| Software | 27 | What software, if applicable, was used to manage the data? |  |
| Participant checking | 28 | Did participants provide feedback on the findings? |  |
| Reporting | | | |
| Quotations presented | 29 | Were participant quotations presented to illustrate the themes/findings? Was each quotation identified? | Direct quotations from participants illustrate the themes and support the conclusions. – Methods  e.g. I often think: 'I can do so much more than what I’m doing now.' I mostly do a lot of administration, a lot of ancillary work […], whereas I would have preferred to use that time to talk longer with my patient. – HCP 20 – Results |
| Data and findings consistent | 30 | Was there consistency between the data presented and the findings? | We strived to maintain coherence between the data showcased and the conclusions by incorporating quotes to bolster our interpretations and findings.  [See Item 29] |
| Clarity of major themes | 31 | Were major themes clearly presented in the findings? |  |
|  |  | End of the Table |  |
