# Supplementary material for: Healthcare professionals’ perspectives on digital biomarkers for monitoring inflammatory arthritis: insights from a qualitative study rooted in design thinking
Source: EULAR Rheumatol Open. 2025 Dec 19;2(1):19–28. doi: 10.1016/j.ero.2025.11.021 (PMC13292230; doi:10.1016/j.ero.2025.11.021)
Supplement: Supplementary file 3 [file mmc3.docx]

**Supplementary Material S3: Individual Patient Characteristics**

| Participant | Occupation | Age Group | Hospital Type | | | Work Experience | | | Personal Experience of Health Monitoring with Wearables | | | | | | | | | | | |
| --- | --- | --- | --- | --- | --- | --- | --- | --- | --- | --- | --- | --- | --- | --- | --- | --- | --- | --- | --- | --- |
| # | R/N | Years | G/A/I | | | Years | | | Smartphone | | | | Smartwatch | | | No | | Comments | | |
| Focus Group 1 – 4 Female \| 1 Male | | | |  | | |  | | |  | | | |  | | | |  |  |  |
| 1 | R | 40s | General | | | 5 | | |  | | |  | | | | | ✓ | Never considered | | |
| 2 | R | 40s | General | | | 6 | | |  | | |  | | | | | ✓ | No need, in good health | | |
| 3 | R | 40s | General | | | 4 | | |  | | |  | | | | | ✓ | Not interested | | |
| 4 | R | 40s | General | | | 12 | | | ✓ | | |  | | | | |  | Pedometer | | |
| 5 | R | 30s | Independent | | | 1 | | |  | | | ✓ | | | | |  | Garmin smartwatch | | |
| Focus Group 2 – 4 Female | | | |  | | |  | | |  | | | |  | | | |  |  |  |
| 6 | N | 50s | Independent | | | 30 | | |  | | |  | | | | | ✓ | No need, in good health | | |
| 7 | N | 40s | General | | | 3 | | | - | | | - | | | | | - | - | | |
| 8 | N | 50s | General | | | 15 | | |  | | | ✓ | | | | |  | Samsung active watch + running app | | |
| 9 | N | 50s | General | | | 20 | | |  | | |  | | | | | ✓ | Nothing important to monitor | | |
| Focus Group 3 – 4 Female \| 1 Male | | | | |  | | |  | | |  | | | |  | | |  |  |  |
| 10 | R | 40s | General | | | 4 | | |  | | |  | | | | | ✓ | No need | | |
| 11 | R | 40s | General | | | 12 | | | ✓ | | |  | | | | |  | Pedometer | | |
| 12 | R | 40s | General | | | 6 | | | ✓ | | |  | | | | |  | Apple Health app | | |
| 13 | R | 30s | Independent | | | 1 | | |  | | |  | | | | | ✓ | No need | | |
| 14 | R | 40s | General | | | 6 | | |  | | |  | | | | | ✓ | Not interested, not very digitally inclined, no need. | | |
| Focus Group 4 – 5 Female | | | | |  | | |  | | |  | | | |  | | |  |  |  |
| 15 | N | 50s | General | | | 26 | | |  | | | ✓ | | | | |  | Garmin smartwatch | | |
| 16 | N | 50s | General | | | 25 | | | ✓ | | |  | | | | |  | Pedometer + Blood pressure monitor | | |
| 17 | N | 50s | General | | | - | | | ✓ | | |  | | | | |  | Pedometer | | |
| 18 | N | 50s | General | | | 3 | | |  | | | ✓ | | | | |  | Running watch | | |
| 19 | N | 30s | Academic | | | 6 | | |  | | |  | | | | | ✓ | No need, assesses fitness intuitively | | |
| Focus Group 5 – 2 Female \| 4 Male | | | | |  | | |  | | |  | | | |  | | |  |  |  |
| 20 | R | 60s | General | | | 23 | | |  | | |  | | | | | ✓ | No need | | |
| 21 | R | 50s | General | | | 11 | | | ✓ | | |  | | | | |  | Pedometer | | |
| 22 | R | 40s | General | | | 11 | | |  | | | ✓ | | | | |  | Smartwatch | | |
| 23 | R | - | General | | | 11 | | | - | | | - | | | | | - | - | | |
| 24 | R | 50s | General | | | 5 | | |  | | |  | | | | | ✓ | - | | |
| 25 | R | 40s | Academic | | | 5 | | |  | | | ✓ | | | | |  | Applewatch | | |
| Continue on next page | | | | | | | | | | | | | | | | | | | | |
| Participant | Occupation | Age Group | Hospital Type | | | Work Experience | | | Personal Experience of Health Monitoring with Wearables * | | | | | | | | | | | |
| # | R/N | Years | G/A/I | | | Years | | | Smartphone | | | | Smartwatch | | | No | | Comments | | |
| Focus Group 6 – 1 Female \| 2 Male | | | | |  | | |  | | |  | | | |  | | |  |  |  |
| 26 | R | 60s | Academic | | | 23 | | | ✓ | | |  | | | | |  | Weight watching app | | |
| 27 | R | 60s | Academic | | | 21 | | |  | | | ✓ | | | | |  | Garmin smartwatch + app | | |
| 28 | R | 40s | General | | | 5 | | |  | | |  | | | | | ✓ | No need, assesses fitness intuitively | | |
| Interview 3 – 1 Male | | | | |  | | |  | | |  | | | |  | | |  |  |  |
| 29 | ID NOT ASSIGNED | | | | | | | | | | | | | | | | |  | | |
| 30 | R | 50s | General | | | 17 | | | ✓ | | |  | | | | |  | Running app | | |
| Focus Group 7 – 3 Female \| 2 Male | | | | |  | | |  | | |  | | | |  | | |  |  |  |
| 31 | R | 60s | General | | | 27 | | |  | | | ✓ | | | | |  | Garmin smartwatch | | |
| 32 | R | 30s | General | | | 1 | | |  | | | ✓ | | | | |  | Garmin smartwatch, for exercising | | |
| 33 | R | 40s | Academic | | | 7 | | |  | | |  | | | | | ✓ | No need, does not like it | | |
| 34 | R | 30s | Academic | | | 8 | | |  | | | ✓ | | | | |  | Garmin smartwatch | | |
| 35 | R | 30s | General | | | 3 | | |  | | |  | | | | | ✓ | Does not like it, prefers intuitive fitness monitoring | | |
| End of the Table | | | | | | | | | | | | | | | | | | | | |
| ^*^ HCPs reported their personal experiences with digital health/fitness monitoring. Smartwatch usage implies the usage of a smartphone app to review the collected data. Smartphone numbers indicate usage of smartphone apps without the usage of a smartwatch. | | | | | | | | | | | | | | | | | | | | |
