# Supplementary material for: Healthcare professionals’ perspectives on digital biomarkers for monitoring inflammatory arthritis: insights from a qualitative study rooted in design thinking
Source: EULAR Rheumatol Open. 2025 Dec 19;2(1):19–28. doi: 10.1016/j.ero.2025.11.021 (PMC13292230; doi:10.1016/j.ero.2025.11.021)
Supplement: Supplementary file 4 [file mmc4.docx]

**Supplementary Material S4: Quotes and Translations**

| Quote NL | Quote Translation EN | | Participant ID | | |
| --- | --- | --- | --- | --- | --- |
| Theme 1: The contemporary landscape of rheumatology care | | | | | |
| We zitten nu in een enorme luxe positie. De huidige zorg is kwalitatief hartstikke goed, maar niet houdbaar. Ik denk ook dat er dat we daar heel erg naar toe moeten om te kijken; "waar zijn wij nou echt voor nodig ?[…]" En zoals we de reumazorg nu hebben ingericht, doen we ook wel heel veel controles zonder dat dat consult bij mij toegevoegde waarde heeft […], maar omdat we dat gewend zijn. | | We are currently in an incredibly privileged position. The current standard of care is of very high quality, but it’s not sustainable. I also think we really need to start looking: 'what are we truly needed for? […]' And the way we’ve organized rheumatology care now, we do a lot of check-ups, where the consultation with me doesn’t really add value […], but we do them because we’re used to it. | HCP 28 | | |
| Dat is meer een stukje aanzien: “Als de dokter het zegt dan neem ik het in en dan zie ik wel hoe het loopt." Terwijl ik bij de jongere generatie, mensen ook echt heel kritisch zie kijken en de bijsluiters hebben gelezen en gegoogled hebben van wat het allemaal voor effecten kan hebben. En dan zeggen van: "goh, ik wil dat toch wel eens over hebben.” | | That’s more about authority: 'If the doctor tells me to, I’ll take it and see how it goes.' Whereas with the younger generation, I really see people looking critically, having read the leaflets and googled what effects it might have. And then they say: 'hmm, I’d actually like to talk about that. | HCP 18 | | |
| Ik denk heel vaak: “ik kan veel meer doen dan wat ik nu doe.” Ik doe vooral veel administratie, heel veel oneigenlijk werk […], terwijl ik eigenlijk die tijd had willen gebruiken om langer met mijn patiënt te praten. | | I often think: 'I can do so much more than what I’m doing now.' I mostly do a lot of administration, a lot of ancillary work […], whereas I would have preferred to use that time to talk longer with my patient. | HCP 20 | | |
| Ik denk dat er misschien een verschuiving komt. […]. Dat je misschien die stabiele mensen wat meer op afstand kan zien of door een andere zorgprofessional […] en dat je zelf wat meer kan focussen op de mensen die complexer zijn, die misschien iets meer tijd en aandacht nodig hebben. | | There might be a shift coming. […]. Where maybe we can monitor stable patients more remotely or through another healthcare professional […] and that we can focus more on the people who are more complex, who might need a bit more time and attention. | HCP 5 | | |
| Theme 2: Limitations of best-practice digital care approaches | | | | | |
| Daar zit een categorie mensen die gewoon wat accepteren qua verdikkingen. […] Dat maakt het niet altijd even makkelijk om het dan op afstand te beoordelen. […]. Wij moeten echt voelen. Als het echt dik is, dan zie je dat wel, maar bij de echt subtiele, nee, dat valt tegen. | | There’s a group of people who just accept some swelling. […] That doesn’t always make it easy to assess it remotely. […]. We really need palpation. If it’s really swollen, you can see it visually, but with subtle ones.. No, that’s difficult. | HCP 22 | | |
| Je kan dat dan niet zien. […]. Dan ben je natuurlijk wel een beetje afhankelijk van wat er iemand dan aangeeft. Hè, want het gebeurt inderdaad ook wel dat mensen zeggen dat ze zwelling hebben en dat je dan inderdaad uitnodigt op de poli en dan blijkt het dus niet te zijn. | | You can’t see it. […]. So you’re kind of dependent on what the person reports. Because it does happen that people indicate they have swelling, and then you invite them to the clinic and it turns out they don’t. | HCP 16 | | |
| Het menselijke het echt interesse tonen in de patiënt. Dat vind ik zelf echt wel lastiger als ik iemand bel en […] hoe je wordt vertrouwd, hangt daar ook van af. Voor de langere termijn is dat ook je slagen van je therapie […] als ze uiteindelijk vertrouwen in je hebben en doen wat je ze adviseert. | | The human aspect—genuinely showing interest in the patient. I personally find that more difficult when I’m calling someone and […] how you’re trusted also depends on that. In the long term, it also affects the success of your therapy […] if they eventually trust you and follow your advice. | HCP 2 | | |
| Continue on next page | | | | | |
| Quote NL | Quote Translation EN | | Participant ID | | |
| Theme 2 - Continued | | | | | |
| Maar wat ik wel heb gemerkt dat je bij telefonische consulten toch wel minder geneigd bent om dynamisch je medicatie aan te passen, met name het afbouwen bijvoorbeeld. Omdat je toch een beetje denkt van: "nou ja, het gaat goed, ja, we houden het zo." Als je iemand fysiek ziet ben je sneller geneigd te zeggen van: "nou gaat goed, volgende keer merk ik het vanzelf wel.” | | What I’ve noticed is that during telephone consultations, you’re less inclined to adjust medication dynamically – especially to taper it, for example. Because you tend to think: “well, it’s going fine, let’s just keep it as is.” When you see someone in person, you’re more likely to say: “it’s going well, I’ll notice next time if anything changes.” | HCP 22 | | |
| Theme 3: Possible opportunities of technology | | | | | |
| De vraag is of je het op andere manieren kan meten. […]. Dus dat je kan zien of iemand een actieve reuma heeft aan bijvoorbeeld hoeveel of hoe hard ze lopen. En als daar een afwijking in komt dat je op hele andere manieren gaat denken over hoe je reuma meet en activiteit meet. | | The question is whether you can measure it differently. […]. So that you can see whether someone has active disease based on, e.g, how much or how fast they walk. And if there’s a deviation, you start thinking in completely different ways about how to measure IA and disease activity. | HCP 31 | | |
| De druk is om uit enorme hoeveelheid data over mensen de juiste dingen aan elkaar te koppelen. Misschien moeten we één gegeven van de Albert Heijn koppelen aan één ding uit een sensor, één ding uit een biometrische variabele en één ding uit een vragenlijst, en dan krijgen we een compleet beeld van wat er gebeurt. | | The pressure is to connect the right pieces of information from a huge amount of data about people. Maybe we need to link one piece of data from Albert Heijn to one thing from a sensor, one biometric variable, and one item from a questionnaire, and then we get a complete picture of what’s going on. | HCP 30 | | |
| Ik vind het idee van #34 zo leuk: ' dan kan je smartwatch... ' Ze zagen al van te voren aankomen waar de flare kwam. […] Dat je dan ziet dat die waarde oploopt, ook als patiënt in je eigen dashboard. Dat die dan denkt: '[…] ik moet nu met mijn reumatoloog gaan overleggen wat er nodig is.' En dan zien we patiënten eigenlijk alleen op momenten dat er echt therapie veranderingen moeten zijn. Als ik het zo hoor vond ik dat wel een heel mooi beeld, dat er iets is wat patiënt is gewoon regelmatig hoogfrequent met iets wat weinig tijd kost meet. | | I really like the idea from #34: ‘that your smartwatch can..’. They could already see in advance where the flare was coming. […] That you can see a value rising, even as a patient in your own dashboard. And then the patient could think: ‘[…] I need to consult my rheumatologist about what’s needed.’ And then we would only see patients at moments when therapy actually needs to be adjusted. Hearing it like that, I thought it was a really nice vision; that there is something that measures the patient regularly, frequently, and with minimal effort. | HCP 31 | | |
| Als je al iets weet over ziekte activiteit voordat iemand binnenkomt, dan kun je iets sneller tot de kern komen. Dat maakt het gesprek denk ik wel wat inhoudelijker. […]. Is het actieve ziekte waar we mee aan de slag moeten, of is er wat anders aan de hand? En zo denk ik dat je sneller een patiënt met medicatie dan wel andere tips hopelijk wat beter krijgt. | | If you already know something about disease activity before someone comes in, you can get to the core a bit faster. I think that makes the conversation more substantive. […]. Is it active disease we need to address, or is something else going on? And that way, you could help a patient get better more quickly, whether through medication or other advice. | HCP 35 | | |
| Als iemand […] zegt: ‘Ik kan nog geen 10 meter lopen, dan vind ik het altijd bijzonder dat ze dan wel gewoon de poli af kunnen lopen. Maar dan kun je natuurlijk wel laten zien van: “Ja maar je hebt toch zo veel stappen gezet.” Dat kan wel interessant zijn. | | When someone says: ‘I can’t even walk 10 meters,’ I always find it remarkable that they can still walk out of the clinic. But then you can show them: “Yes, but you’ve taken quite a few steps.” That can be interesting. | HCP 8 | | |
| Continue on next page | | | | | |
| Quote NL | Quote Translation EN | | Participant ID | | |
| Theme 3 - Continued | | | | | |
| Ik denk dat technologie ons kan ondersteunen omdat de patiënt door de technologie wordt ondersteunt en wordt herinnert aan: het is weer tijd om je bloed te laten controleren, je pillen te bestellen, etc […]. Technologie kan ons dan ook helpen in het bieden van informatie on demand voor de patiënt. | | I think technology can support us because it supports the patient and reminds them: it’s time to get your blood checked again, to order your pills, etc. […]. Technology can also help us provide on-demand information for the patient. | HCP 28 | | |
| Theme 4: Concerns about digital remote monitoring | | | | | |
| Daar zit dan wel mijn zorg; als alles veel meer meetbaar enzovoort wordt, hoe blijf je oog houden wie die persoon is? – HCP 33. Ik denk dat misschien de persoonlijkheid […] er een beetje af gaat. Dat iemand een beetje onhandig naar binnen komt of iemand heeft een rare jas aan, dat zie je dan dus allemaal niet. […]. Terwijl het je wel iets geeft over het karakter van de patiënt waar je later misschien iets aan hebt als het moeilijker gaat. | | That’s where my concern lies; if everything becomes much more measurable and so on, how do you keep sight of who a person is? – HCP 33. I think maybe the personality […] gets a bit lost. Someone might come in a bit awkwardly or be wearing a strange coat, you don’t see any of that. […]. Yet it tells you something about the patient’s character, which might be useful later on when things get more difficult. | HCP 32 | | |
| Een nadeel zou in mijn optiek kunnen zijn van; in hoeverre zijn mensen dagelijks dan constant bezig met hun chronische aandoening? – HCP 9. Als ie een keer een luie dag houdt, dan registreert die stappenteller dat. […]. Is dat dan een stimulans of zullen mensen zich misschien schamen? | | A downside, in my view, could be: to what extent will people be constantly focused on their chronic condition? – HCP 9. If they have a lazy day for once, the step counter records that. […]. Is that a motivator, or might people feel ashamed? | HCP 17 | | |
| Hoe vaak kom ik tegen dat ze hun medicijnen toch niet goed gebruiken. […] Ik kom het bij iedereen tegen, juist ook de ervaren die denken: "nou, ga ik zelf dokteren." Maar ook de nieuwere mensen die het niet snappen. | | How often I encounter that it turns out people don’t use their medication properly. […] I see it in everyone, even the experienced ones who think: “well, I’ll just doctor myself.” But also the newer patients who don’t understand it. | HCP 18 | | |
| Ik denk wel dat het ons meer inzichten gaat geven. De vraag is alleen, ik zie daar ook wel werkverschaffing. Want we moeten die data natuurlijk ook doornemen, we moeten het ook goed kunnen interpreteren. Wie gaat dat doen en hoe kunnen we de fouten eruit halen. […] Iedere technologie is natuurlijk een foutgevoeligheid. Als er ineens hoge scores zijn terwijl er iets verkeerd doorgegeven wordt, dan schieten wij in de stress […]. Hoe ga je met dat soort dingen om? | | I do think it will give us more insights. The question is, though – I also foresee it creating more work. Because we have to go through the data, and we also have to be able to interpret it properly. Who’s going to do that, and how do we filter out the errors? […] Every technology is prone to errors. If suddenly there are high scores because something was transmitted incorrectly, we get stressed […]. How do you deal with things like that? | HCP 13 | | |
| Theme 5: Factors influencing the adoption of digital remote monitoring | | | | | |
| Ik denk dat het vergelijkbaar is met het overgaan naar het elektronisch dossier van een papieren dossier. Ja, tuurlijk waren daar sommigen enthousiast over en sommigen heel lang aan het tegenstribbelen, maar nu werken we allemaal met computers, niet meer met een decursus blad. Dus ik denk, natuurlijk zijn er mensen die voorop lopen en andere mensen die het liefst iets bij het oude houden, maar ik denk dat we uiteindelijk toch allemaal die kant op moeten bewegen. | | I think it is comparable to transitioning from paper records to electronic records. Yes, of course, some were enthusiastic about it, while others resisted for a long time, but now we all work with computers instead of handwritten progress sheets. So naturally, some people lead the way while others prefer to stick to tradition, but ultimately, I believe we all have to move in that direction. | HCP 5 | | |
| Continue on next page | | | | | |
| Quote NL | Quote Translation EN | | Participant ID | | |
| Theme 5 - Continued | | | | | |
| Eerst moet ik weten, welke soort informatie wij gaan krijgen? […] Zijn het predictoren van een flare? En wat is de echte evidence. En misschien krijg ik een alert in HIX; ‘Meneer X heeft een kans van 80% op een flare binnen de komende twee weken.’ Ja, en nu? | | First, I need to know what kind of information we will be receiving. […] Are these predictors of a flare? And what is the actual evidence? Perhaps I will receive an alert in HIX: ‘Mr X has an 80% chance of a flare in the next two weeks.’ Right. But then what? | HCP 34 | | |
| Tot nu toe zijn de cijfers over wat de smartphone kan meten – die worden vooral geleverd door de fabrikant en dan is het natuurlijk geweldig. Wij worden waarschijnlijk opgezadeld met een HIX-achtig computer model dat gevalideerd is door een of andere ingenieur, en dat wordt ons gewoon ‘Hier als je blieft, doe het maar. Het is harstikke goed het is getest op 30 patiënten en het deed het gewoon.’ [sarcastisch] | | So far, the figures regarding what smartphones can measure have largely been provided by the manufacturers, and – of course – those look impressive. But we will likely be handed a HIX-style computer model, validated by some engineer, and simply told: ‘Here you go, use it. It’s absolutely fantastic. It was tested on 30 patients Wand worked just fine.’ [Sarcastic] | HCP 26 | | |
| Ik denk waar de meeste mensen behoefte aan hebben […] is als er wat is, dat ze aan de bel kunnen trekken en dat ze dan ook gelijk gezien kunnen worden. En als je die vangnetten biedt, dan weten ze ‘ik durf wat langer weg, maar als er wat is dan kan ik altijd gezien worden.’ | | I think what most people need […] is the ability to raise the alarm if something happens and be seen straight away. And if you provide those safety nets, they’ll know: ‘I feel comfortable being away longer, but if something happens, I can always be seen.’ | HCP 25 | | |
| Niet iedereen is ook digitaal even onderlegd hè, en dan maakt het niks uit of iemand 35 of 80 is. […] We hebben het natuurlijk ook over laaggeletterden. – HCP 6. Inderdaad lang niet alle patiënten zijn geschikt. Ten eerste moet iemand natuurlijk goed begrijpen of de klachten te maken hebben met inflammatie of niet, ze moeten zelf ook ziekte inzicht hebben en ook is de ene RA de ander niet. […] Het is lastig om die allemaal op gestandaardiseerde manier te volgen via de digitale weg. | | Not everyone is equally proficient with digital tools, and whether someone is 35 or 80 makes no difference. […] We are also considering people with low literacy levels. – HCP 6. Indeed, not all patients are suited to this. First, they must be able to understand whether their symptoms are due to inflammation or something else; they need to have insight into their condition, and one case of RA is not the same as another. […] It is difficult to monitor everyone in a standardised way via digital methods. | HCP 5 | | |
| Ik denk dat vooral geldt als je een populatie hebt die je goed kent. Ik heb nu ervaren dat ik een deel van de populatie overneem van een collega met mensen die ik absoluut nooit eerder heb gezien, dan heb je natuurlijk totaal geen gevoel bij een persoon en andersom ook niet. Dan mis je dus die mensen die nooit klagen en die dan vervolgens inderdaad helemaal actief weer een keer binnen komen. | | I think this is especially relevant when dealing with a familiar patient population. I recently took over part of a colleague’s patient group, some of whom I had never encountered before. Naturally, I have no personal sense of them, and vice versa. That means you will overlook those patients who never complain but then suddenly turn up, fully active once again. | HCP 5 | | |
| End of the Table | | | |  |  |
